# Arsenic Plus Alcohol as a Cause of Neuritis

**Published:** 1901-01-19

**Authors:** 


### Hospital Clinics and Medical Progress.

### ARSENIC PLUS ALCOHOL AS A CAUSE OF NEURITIS.

By flux of time it has become possible to correlate the various observations made during the recent outbreak of arsenical poisoning in Lancashire and the Midlands, and to compare them with those made at various periods in regard to other sources of arsenical poisoning and other causes of neuritis. From the descriptions given of the Manchester outbreak, and from the definite proofs offered of the presence of arsenic in the materials used in brewing and even in the beer itself, no one can doubt that arsenic has been at the bottom of the mischief; yet when we put together all the facts at our disposal the matter appears by no means so simple as it did when the cry first came from the manufacturing districts that the people were being poisoned by arsenic in beer.

*Omne ignotum pro magnifico.* When people heard only the crude assertion that there was arsenic in the beer, and when no one knew how much, the conclusion was natural that the amount taken must have been considerable, and at least amply sufficient to account for the symptom observed. But with time has come exactitude. We know now that the quantity taken has in many cases been but small, and, as we pointed out some time ago, that far larger doses of arsenic have frequently been administered medicinally without any evil consequences. At the meeting of the Royal Medical and Chirurgical Society last week Sir William Gowers and Dr. Buzzard followed in the same strain, pointing out, as we had done, that in cases of epilepsy far larger doses than most of those recorded at Manchester had often been taken, and had been continued during far longer periods, without injury. The conclusion then seems irresistible that some other influence besides the arsenic has been at work: perhaps that alcohol and arsenic together act much more injuriously than either alone; perhaps that the arsenic contained in beer brewed from arseniated products exists in organic combination and in a much more active form than the crude salts from which our knowledge of doses is derived. Ever since symmetrical peripheral neuritis began to be clinically recognised it has been apparent that the cause of the disease must be some kind of poison, and it is now generally accepted that the degenerative changes included under that designation may be produced by lead, arsenic, mercury, &c.; by alcohol, ether, bisulphide of carbon, and other such compounds; by the toxins produced by many pathogenic organisms, such as those of diphtheria, influenza, pneumonia, and syphilis; and also by the abnormal products of metabolism developed in various morbid states, as, for example, in gout; and it has become a common practice with clinicians to make use of an ætiological classification founded upon the presumed cause of the malady in any given case. Hence we hear of "gouty neuritis," "alcoholic neuritis," and "arsenical neuritis." The experience of this epidemic seems to suggest, however, that in seeking for causes we must in future not be content to study the effects of isolated poisons, but must have regard to the grouping of causes, the coincidence in any given case of several influences each more or less efficient, and must inquire whether by aid of such groupings and coincidences very small doses of some of these toxic agents—doses so small as to appear harmless by themselves—may not be so reinforced by association as to become potent for evil. There is a manifest inclination among some of the Manchester observers to throw doubt upon the hitherto accepted descriptions of alcoholic neuritis. It has long been observed, at any rate in Manchester, that the form of neuritis attributed to alcohol is far more common among beer drinkers than among those who indulge in spirits, and not unnaturally it is now suggested that "alcoholic neuritis" has been a mistake all along, and that arsenic has been at the root even of the so-called alcoholic cases. We do not think that this is a proposition that can be maintained. Possibly it may be true of a certain proportion of the older cases, as it certainly is of many of the more recent ones, that what was at first put down to alcohol was really due to arsenic; but that does not take away from the fact, well certified by competent observers, that there is such a condition as alcoholic neuritis produced by alcohol alone. On the other hand, it is quite certain that there is such a thing as arsenical neuritis produced by arsenic alone. The strange thing which we have to face is the fact—for it appears to be a fact—which has been brought out by the history of some of the Manchester cases, namely, that symptoms of poisoning of the nervous system may be produced by doses which, whether we have regard to the alcohol or the arsenic, would seem to be quite insufficient to do any harm if we judge by our daily observations as to the quantities of arsenic and of alcohol which may be taken separately without apparent injury.

Thus we come to the hypothesis of mixed toxins, and to the idea that arsenic plus alcohol, and especially plus alcohol in the particular form of beer, may have a specially deleterious influence. It must be confessed that in regard to this we have no real knowledge, but it would be easy to point to many facts in regard to the acute infectious diseases which strongly suggest that mixed toxins greatly aggravate each other's pathogenic influence. The relation between plumbism and gout, especially the gout of the London workman, which is mostly of beery origin, points in the same direction, suggesting that beer plus a metallic poison is a much more injurious influence than either one or the other by itself. These are but speculations. The fact, however, remains, as was pointed out by Sir W. Gowers and Dr. Buzzard, that epileptic and other patients are in the habit of taking far larger quantities of arsenic, and for longer periods of time, than those taken by the beer-drinkers who have suffered from "arsenical" neuritis. Arsenic in itself does not then furnish a complete explanation of all the phenomena observed in this outbreak.

